# Screening for caregivers at risk: Extended validation of the short version of the Burden Scale for Family Caregivers (BSFC-s) with a valid classification system for caregivers caring for an older person at home

**DOI:** 10.1186/s12913-018-3047-4

**Published:** 2018-04-02

**Authors:** Anna Pendergrass, Cintia Malnis, Uta Graf, Sabine Engel, Elmar Graessel

**Affiliations:** 1 Center for Health Services Research in Medicine, Department for Psychiatry and Psychotherapy, Friedrich-Alexander-University Erlangen-Nuremberg (Germany), Schwabachanlage 6, 91054 Erlangen, Germany; 2 The Catholic University of Applied Sciences of North Rhine-Westphalia Department Social Services, Campus Paderborn (Germany), Leostraße 19, 33098 Paderborn, Germany

**Keywords:** Informal caregivers, Caregiver burden, Questionnaire, Short version of the burden scale for family caregivers, Validation

## Abstract

**Background:**

Informal caregivers’ (CGs’) subjective burden is an important aspect of the care situation because it is linked to various outcomes such as health, mortality risk, institutionalization, and caregiving style. The aims of this study were a) to examine the convergent and discriminant validity of the 10-item short version of the Burden Scale for Family Caregivers (BSFC-s) and b) to develop a valid classification system for interpreting BSFC-s scores.

**Methods:**

In this cross-sectional study, we analyzed data obtained from 386 informal CGs who applied for an initial grade or upgrade of the care level for the care recipient at the Medical Service of Compulsory Health Insurance Funds of Bavaria (Germany). To validate the BSFC-s, we analyzed the reliability and the convergent/discriminant validity. We calculated correlations with the short form of the Giessen Symptom Complaints List (GBB-24), the Caregiver Strain Index (CSI), the personal further development sub-scale of the Berlin Inventory of Caregivers’ Burden with Dementia Patients (BIZA-D), and other scales for establishing informal CGs’ situations. To develop the classification system, we compared the percentile ranks of the GBB-24 with the respective BSFC-s sum scores and their distributions and derived three classification categories.

**Results:**

Results confirmed the convergent and discriminant validity of the BSFC-s (GBB-24: *r* = 0.68; CSI: *r* = 0.70; BIZA-D: *r* = 0.16; *p* < 0.001). For informal CGs with low subjective burden, the risk of physical psychosomatic complaints was elevated to a less than average level (BSFC-s scores of 0-4). In those with a moderate subjective burden (BSFC-s scores of 5-14), the risk was elevated. In those with a high burden (BSFC-s scores of 15-30), the risk was substantially elevated.

**Conclusions:**

The BSFC-s is a valid scale for measuring subjective burden in informal CGs. The risk of physical psychosomatic complaints, which is a consequence of subjective CG burden, can be determined by using the valid classification system to deduce the necessity for action and to give concrete recommendations for interventions. The BSFC-s should therefore be employed as a screening instrument in medical contexts and in counseling services for informal CGs.

## Background

“Subjective burden” is defined as a person’s subjective self-evaluation of feeling burdened. Many previous studies on informal caregivers (CGs) have focused on this variable because it is associated with many important outcomes of the CGs, the care recipients (CRs), and the overall care situation [1]. Particularly, caregivers of an older adult report a higher burden than other caregivers [2].

A meta-analysis conducted by Pinquart and Sörensen [3], for example, showed that the impairment of informal CGs’ physical health increases with the extent of their subjective burden. It has also been demonstrated that if informal CGs are differentiated by their level of burden, CGs with a higher level of burden demonstrate a higher risk of mortality [4]. In their review of abusive behavior toward older persons with dementia, Boye and Yan [5] found that in several studies, CGs’ subjective burden was seen as a risk factor for abusive behavior in the form of physical or psychological violence. Thus, the risk for abusive behavior toward CRs increases as the informal CGs’ perceived burden increases. Eska, Graessel, Donath, Schwarzkopf, Lauterberg and Holle [6] found various different predictors of institutionalization in people with dementia. Here again, alongside age, level of education, and other factors, CGs’ subjective burden was a significant predictor of institutionalization. The CR’s likelihood of being institutionalized can therefore be expected to increase with the severity of the CG’s subjective burden. This corroborates the health-economic relevance of the construct of “subjective caregiver burden.”

Various validated questionnaires for assessing this construct have been used in international research (e.g. the Zarit Burden Interview [7], the Caregiver Strain Index [8], and the CarerQol [9]). The Burden Scale for Family Caregivers (BSFC-s) was developed for German-speaking countries [10] and is available free of charge in over 20 languages (www.caregiver-burden.eu). This scale consists of a total of 28 items and is based on Lazarus and Folkman’s [11] transactional stress model. This model suggests that the perception of stress depends primarily on cognitive processes such as the primary and secondary appraisals of a situation. In the case of informal CGs, the perceived burden thus depends on how CGs assess the situation itself and their own ability to meet these demands [12]. The scale is intended to measure specifically the “stress” that arises from the caregiving situation. The higher the score, the greater the burden. This scale has shown positive results on all psychometric quality criteria investigated to date [13] and has been validated in large samples and in several languages (e.g. Turkish [14] and Danish [15]).

To provide a quick and economical way to assess subjective burden, Graessel, Berth, Lichte and Grau [16] developed and validated a short form of the BSFC (BSFC-s) consisting of 10 items. This first validation study on the short version (BSFC-s), which assessed amongst other variables the correlations between the BSFC-s score and scales measuring the severity of cognitive impairment, the severity of disturbing behavior and the diagnosis of a depressive episode concerning the caregiver, showed that it measures the construct as well as the long version does, without any loss of quality [16]. This previous validation study investigated both the internal consistency (Cronbach’s alpha of 0.92 for the complete scale) and the construct validity (e.g. moderate correlation with disturbing behaviour of the care receiver (rS = .53); higher scores in depressed versus not depressed caregivers (eta = .22) and moderate correlations with care level (eta = .31)) of the scale [16].

To provide an extended validation of the BSFC-s, an additional study of its convergent and discriminant validity is required to build on the results that have been obtained to date. According to DeVon et al. [17] is the convergent validity the correspondence between constructs that are theoretically similar while discriminant validity is on the other hand the capability of a questionnaire to differentiate between constructs that are theoretically different. To determine convergent validity, this present study was designed to establish an association between BSFC-s score and other questionnaires that measure the construct of subjective burden. To determine discriminant validity, the goal was to calculate the associations between the BSFC-s and other scales that measure a different construct.

The second aim of this study was to develop a valid classification system to enable members of various professions in the practice of healthcare (e.g. in general practice or in counseling services for CGs) to interpret the BSFC-s scores more easily and thus to provide them with a meaningful screening instrument. To develop the classification system, we used a common and easy-to-understand external factor that is linked to subjective burden but measures another parameter that is of substantial practical importance. Among the parameters associated with subjective burden mentioned above, the factor “physical health” is highly suitable because physical health on the one hand is considered to be very important by the general population and also because there are norm-referenced questionnaires available for this factor. In developing this classification system, the goal was to be able to use the BSFC-s scores to assess the risk of psychosomatic complaints. This would make it easier to interpret the effects of CG burden in the healthcare setting and derive concrete recommendations for interventions.

## Methods

### Research design

The present study involved a cross-sectional investigation that used data from a written questionnaire survey. The data came from information provided by informal CGs in self-rating scales. Approval for this study was given by the ethics committee of the Medical Faculty of Erlangen-Nuremberg University (registration number 227_14B).

### Criteria for inclusion and exclusion

The data were collected in connection with applications for benefits from nursing care insurance submitted to the Medical Review Board of the German Statutory Health Insurance Funds (GSHIF). Persons covered by statutory health insurance in Bavaria (Germany) applied to have their claims classified for nursing care benefits in accordance with the provisions of Vol. XI of the German Social Code. In these cases, the GSHIF must visit the applicant at home in order to establish the extent of the need for nursing care. The extent of benefits that the insured persons subsequently receive depends on the result of this assessment. Therefore, to qualify for this study, the person had to be a resident of Bavaria, had to be covered by statutory health insurance, and had to have submitted an application for an initial assessment or an upgrade in the level of nursing care for which they were eligible. Everyone, who was visited by the GSHIF received the questionnaire. There were no restrictions based on type of disease. A total of 1700 questionnaires were distributed over a period of 9 months, and 452 (26.6%) were returned. By voluntarily returning the questionnaire, the participants consented to the anonymous use of their data. A total of 66 persons in this sample were excluded from the statistical analysis. The reasons for the exclusions were, for example, the age of the care recipient (< 64 years, *N* = 53) or too many missing values because the questionnaire had been only partially (< 50%) filled out (*N* = 13).

### Participants

The calculations are based on a sample of 386 informal CGs from all parts of Bavaria who were caring for an elderly person at home. The participants had an average age of 61.3 years (*SD* = 12.2), and 76% of them were women. More information about sample characteristics is given in Table 1.

**Table 1:** Characteristics of the informal caregivers

Characteristic	Total (*n* = 386)
Age, M (SD)	61.3 (12.2)
Women, number (%)	295 (76.4)
Gainfully employed, number (%)	157 (40.7)
Highest educational qualification, number (%)	
University degree	39 (10.1)
Advanced school-leaving examination (*Abitur*)/technical *Abitur*	32 (8.3)
“Realschule” (Middle School)	201 (52.1)
Primary school/“Hauptschule” (lower secondary school)	112 (29.0)
No school-leaving qualification	2 (0.5)
Relationship (to care recipient is …), number (%)	
Father/Mother	188 (48.7)
Father-/Mother-in-law	46 (11.9)
Spouse or life companion	136 (35.2)
Other relative	12 (3.1)
Not a relative (friend, acquaintance, neighbor)	4 (1.0)
Living together, number (%)	270 (69.9)
Duration of care in months, M (SD)	52.8 (51.5)
BSFC-s, M (SD) (range: 0 to 30)	16.1 (7.8)
CarerQol, M (SD) (range: 0 to 14)	7.9 (2.8)
CSI, M (SD) (range: 0 to 13)	7.4 (2.8)
PHQ-9, M (SD) (range: 0 to 27)	8.0 (5.1)
GBB-24, M (SD) (range: 0 to 96)	27.2 (17.1)
BIZA-D, M (SD) (range: 0 to 20)	12.3 (3.8)
ADLs, M (SD) (range: 0 to 17)	3.1 (2.2)
IADLs, M (SD) (range: 0 to 17)	3.4 (2.2)

Note. *M* mean, *SD* standard deviation, *range* the possible ranges of each scale, *BSFC-s* short version of the Burden Scale for Family Caregivers, *CSI* Caregiver Strain Index, *CarerQol* Care-related Quality of Life instrument, *BIZA-D* Berlin Inventory of Caregivers’ Burden with Dementia Patients, *PHQ-9* Depression module of the Patient Health Questionnaire, *GBB-24* Giessen Subjective Complaints List (short form), *ADLs* Activities of Daily Living, *IADLs* Instrumental Activities of Daily Living

### Measures

#### Short version of the burden scale for family caregivers (BSFC-s)

The short version of the Burden Scale for Family Caregivers (BSFC-s) is a 10-item instrument for measuring subjective burden in informal CGs. Each item is a statement that is rated on a 4-point scale with the values “strongly disagree” (0), “disagree” (1), “agree” (2), and “strongly agree” (3). A high degree of agreement indicates higher subjective burden for the caregiver. The reliability (Cronbach’s alpha) is 0.92 [16].

#### Giessen subjective complaints list short form (GBB-24)

The Giessen Subjective Complaints List GBB-24 [18] is a standardized scale for measuring 24 physical complaints in terms of the whether they are fully or partly psychosomatically induced. It contains the four sub-scales “physical exhaustion,” “stomach complaints,” “pain in the limbs,” and “heart complaints,” each of which consists of 6 items. Respondents can then rate their impairment as “not at all” (0), “hardly” (1), “somewhat” (2), “considerable” (3), or “yes, absolutely” (4). A sub-scale score between 0 and 24 points is calculated for each of these areas. An overall score with a range of 0 to 96 points is then computed for “pressure of complaints.” Higher scores indicate more severe “pressure of complaints.” Cronbach’s alpha is 0.94 for the overall score and between 0.82 and 0.88 for the individual sub-scales [18].

#### Caregiver strain index (CSI)

The Caregiver Strain Index (CSI) consists of 13 items and was constructed to measure strain in informal CGs [8]. Each item is a statement that can be responded to with either “no” (0). or “yes” (1) The score can thus lie between 0 and 13. Higher values indicate a higher level of strain in the informal CG. The reliability (Cronbach’s alpha) is 0.86 [8].

#### Berlin inventory of caregivers’ burden with dementia patients (BIZA-D)

The Berlin Inventory of Caregivers’ Burden with Dementia Patients (BIZA-D) measures the subjective and objective burden of informal CGs of persons with dementia [19]. The original measure consists of 20 sub-scales with a total of 88 items. In our survey, we employed the items from the “Personal Further Development” sub-scale, which corresponds to the dimension “Subjectively perceived conflicts between needs and positive aspects of care.” This sub-scale consists of 5 items rated on a 5-point response scale from “never” (0) to “always” (4) and the items are not specifically formulated for caregivers of care receivers with dementia. The reliability of this sub-scale is α = 0.87 [19].

### Care-related quality of life instrument (CarerQoL)

The CarerQoL was developed to measure quality of life in informal CGs in relation to their caregiving activities [9]. The first part consists of 7 items that measure subjective burden that arises from informal care. The statements are rated on a 3-point scale with the severities “no,” “some,” and “a lot.” Higher values thus indicate less strain (better quality of life). The second part consists of an item that asks about the person’s well-being and is measured via a visual analogue scale. This additional item was not used in the present study.

#### Patient health questionnaire (PHQ-D)

The Patient Health Questionnaire (PHQ-D) is a questionnaire that asks about various psychological disorders derived from the DSM-IV criteria [20]. This questionnaire can be employed to support diagnosis and to assess the severity of the person’s illness. One component of the PHQ-D is the depression module (PHQ-9), which consists of 9 questions that check for symptoms of depression. The respondent is asked to rate the frequency of occurrence of these symptoms on a 4-point scale from “not at all” (0) to “nearly every day” (3). In the present study, only the PHQ-9 depression module was administered. The reliability of the depression module is alpha = 0.89 [21].

#### Other variables

The last section of the questionnaire asked for personal details about the CG such as date of birth, sex, level of education, current employment, and relationship to the CR. This part of the questionnaire also asked for the CR’s level of nursing care benefits and the cause of the need for care. Then, the two domains of activities of daily living (ADLs) (according to Mahoney and Barthel [22]) and instrumental activities of daily living (IADLs, according to [23]) were each investigated with one item each. The CG was asked to state whether the CR regularly needed help with certain activities. The first item covered the activities of “getting dressed, personal hygiene, bathing, walking, eating, and going to the toilet” (ADLs) and the second “going shopping, preparing meals, taking medications, running the household, handling financial or other affairs, going on journeys, accompanying (the CR) when going out” (IADLs). The informal CG was requested to answer “yes” or “no” and to enter the average number of hours per day for which the CR needed support in this area.

### Statistical analyses

All calculations were carried out with the IBM SPSS software, Version 21 for Windows. For all analyses, an (alpha) error probability of less than 5% was set to establish the level of statistical significance. Missing values were imputed with the aid of the EM algorithm.

We calculated means and standard deviations for the BSFC-s scores and those of all the other scales.

#### Reliability

In order to determine the internal consistency of the BSFC-s, Cronbach’s alpha was calculated for the overall score. Bortz and Döring [24] recommend an alpha of 0.80 or higher.

#### Validity

In order to test the hypotheses (H) on validity, Pearson’s correlations were calculated between the BSFC-s score and the scores on each of the other scales. According to Mukaka [25], correlations greater than 0.90 are very strong, those between 0.70 and 0.90 are strong, those between 0.50 and 0.70 are moderate, and those between 0.30 and 0.50 are weak. Correlations of less than 0.30 indicate that there is no association.

In order to examine the convergent validity of the BSFC-s, we calculated the correlations between the BSFC-s, the CSI, the CarerQoL, the GBB-24, and the PHQ-9.

H1: Because the CSI and the BSFC-s measure very similar constructs, they were expected to be strongly positively correlated [26].

H2: In the validation study of the CarerQoL, the measure showed a negative correlation with the CSI [9]. High scores on the CarerQoL were thus associated with low values on the CSI. A strong negative correlation was therefore also expected between the CarerQoL and the BSFC-s.

H3: A cross-sectional study showed a positive linear association between the feeling of being burdened by caregiving and the extent of physical complaints [27]. A moderate to strong positive correlation was therefore expected between the GBB-24 and the BSFC-s.

H4: Greater subjective burden is also associated with an increase in depressive symptoms [28]; a moderate or strong positive correlation was therefore also expected between the PHQ-9 and the BSFC-s.

To investigate discriminant validity, we calculated the extent to which subjective burden was correlated with benefits (BIZA-D) and the CR’s degree of independence (ADL and IADL scales).

H5: Initial results indicated no association between subjective burden and the positive aspects of care [29]; thus, no correlation or only a very weak one was expected between the BIZA-D and the BSFC-s.

#### Classification system for interpreting BSFC-s scores

The classification system for the BSFC-s was to be determined by comparing the BSFC-s with a factor of special importance to the individual [30]. Because people’s self-perceived health status is rated very high in the general public, we decided to use the parameter “degree of physical complaints” as an external criterion. It can be measured quickly (only 24 items) and validly with the short form of the Giessen Subjective Complaints List [18]. Also, up-to-date norms are available for the GBB-24. These norms are representative of the population and cover the sample in this study as well.

We chose the classification with three subgroups following other screening instruments in the field of chronical diseases (e.g. the classification of the dementia syndrome via the Mini Mental State Examination [31]). Like the classification system of the long version of the BSFC [32], we formed three groups of burden values that could be interpreted as representing low, moderate, and high levels of burden. In order to divide the scores into three groups, it was necessary to calculate two cut-off values – one value to distinguish between low and moderate burden and the other to differentiate between moderate and high burden. To define these two cut-off values, we followed the 90% rule. The cut-off value between the low and moderate level of burden was specified by the rule that 90% of the low burdened CGs were supposed to have an average or below average risk of physical psychosomatic complaints (percentile rank PR ≤ 50, measured against the age- and sex-specific norms of the GBB). On the other hand the cut-off value between the moderate and high level of burden was also specified by the rule that 90% of the severe caregivers were supposed to have an above-average risk of physical psychosomatic complaints (percentile rank PR > 50).

## Results

The mean values for subjective burden on all three of the scales that we employed (BSFC-s, CSI, and CarerQoL) were approximately in the middle of the respective range. The mean values and standard deviations for these and all other variables are presented in Table 1.

### Reliability

With a Cronbach’s alpha of 0.92, the BSFC-s fulfilled the criterion for a homogeneous scale defined by Bortz and Döring [24].

### Validity

The results confirmed all the hypotheses on the convergent and discriminant validity of the BSFC-s. High values on the BSFC-s were strongly positively correlated with the values on the CSI (H1: *r* = 0.70, *p* < 0.001) and strongly negatively correlated with the values obtained on the CarerQoL (H2: *r* = − 0.72, *p* < 0.001) and. The correlations between the BSFC-s and the GBB-24 (H3: *r* = 0.68, *p* < 0.001) and between the BSFC-s and the PHQ-9 (H4: *r* = 0.68, *p* < 0.001) were also of the same magnitude.

**Fig. 1:**
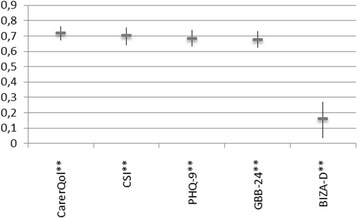
Correlations between the various different scales and the BSFC-s. Note*.* BSFC-s: short version of the Burden Scale for Family Caregivers, CSI: Caregiver Strain Index, CarerQol: Care-related Quality of Life instrument, BIZA-D: Berlin Inventory of Caregivers’ Burden with Dementia Patients, PHQ-9: Depression module of the Patient Health Questionnaire, GBB-24: Giessen Subjective Complaints List (short form)

With regard to discriminant validity, the correlation between the BSFC-s and the BIZA-D was weak (H5: *r* = 0.16, *p* = 0.002). The correlations between the different variables and the BSFC-s and their confidence intervals are shown in Fig. 1.

### Classification system for interpreting the BSFC-s scores

We fulfilled the goals to classify and thus also to define three classes of subjective burden by applying the cut-off values of 5 and 15 (see Table 2).

**Table 2:** Classification system for interpreting the BSFC-s scores

Score on the short version of the Burden Scale for Family Caregivers (BSFC-s)	0 - 4	5 - 14	15 - 30
Frequency in the sample (*n* = 386)	28 (7%)	136 (35%)	222 (58%)
Degree of subjective burden	none to low	moderate	severe to very severe
No. of persons in the class with above-average psychosomatic complaints	10.70%	59.60%	90.50%
Risk of physical psychosomatic complaints	not increased	increased	very much increased

The first class consisted of persons whose BSFC-s scores were between 0 and 4 points. In this range, the great majority (89.3%) of informal CGs were suffering from a degree of physical complaints that was average (PR = 50) or below the population average (PR < 50). For the informal CGs in this class, the risk of having a subjective state of health that was more impaired than the age- and sex-specific norm values for the GBB-24 was very low.

The second class consisted of persons with a BSFC-s score between 5 and 14. In this range, the majority (59.6%) had a degree of physical complaints that was higher than the population average (PR > 50). The informal CGs in this class had an increased risk of having an impaired subjective state of health.

In the third class with BSFC-s scores between 15 and 30, 90.5% of the informal CGs had an above-average degree of physical complaints (PR > 50). The risk of the informal CGs in this class of having an impaired subjective state of health was considerably increased.

## Discussion

The aims of this study were to conduct an extended validation of the short version of the Burden Scale for Family Caregivers (BSFC-s) and to develop a valid classification system for interpreting the scores.

All hypotheses regarding convergent and discriminant validity were confirmed. The strong correlations between the BSFC-s and other international scales measuring strongly related constructs such as the CSI for caregiver strain and the CarerQoL for caregiver Quality of life indicated a high convergent validity of the BSFC-s. The strong correlation between subjective burden and the symptoms of depression (PHQ-9) and psychosomatic complaints (GBB-24) also confirmed the evidence based expectations and indicated a high level of convergent validity.

The very weak correlation between the BSFC-s and the benefits scale provided justification for talking about two independent constructs. It is important to take into consideration that to date, the benefits arising from the care situation and the burden on the informal CGs have been viewed as two ends of one continuum [33]. One example of this is the recommendations for interpretation of the frequently used Zarit Burden Interview [34]. However, the results of our study indicate that burdens and benefits are two different constructs, as presumed by Lloyd, Patterson and Muers [33]. The non-significant correlations between the BSFC-s with the ADL and IADL caregiving items also confirmed our hypotheses about the discriminant validity of the BSFC-s.

**Fig. 2:**
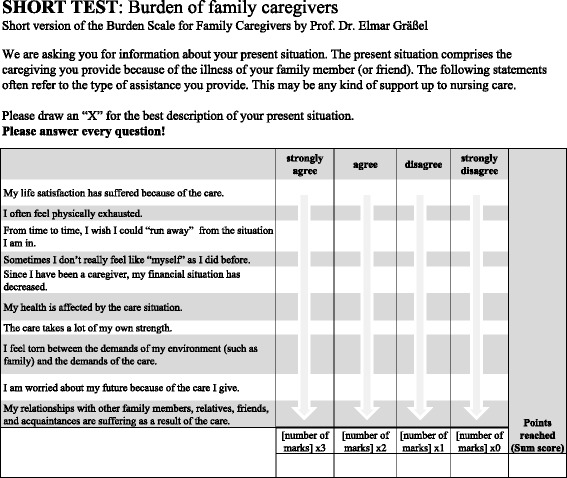
Example of an information brochure. Scale included for the informal caregiver to fill out without assistance

**Fig. 3:**
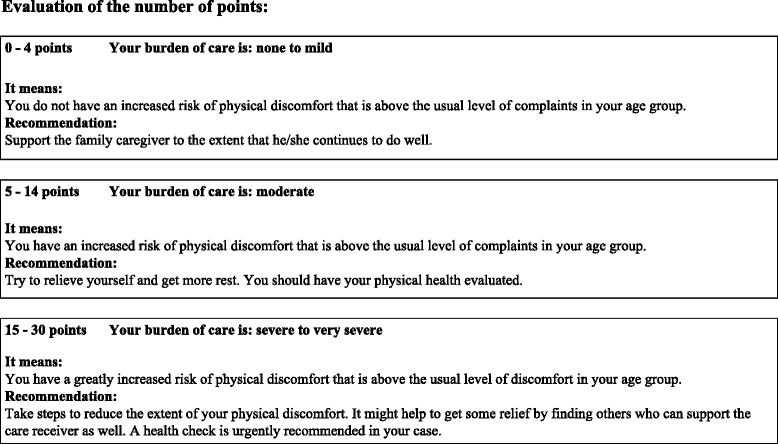
Support for interpreting the results and concrete recommendations to be given to the informal caregiver

An important aspect of research is the transfer of the scientific results into practice [35]. What is particularly interesting in this study is therefore the classification system we developed, as this makes it possible to use the BSFC-s to identify informal CGs with increased levels of health risk. Depending on the level of risk, concrete recommendations for the respective person can be derived. Consequently, interventions to reduce the burden can be delivered before avoidable health problems arise. In this way, it may be possible to prevent or at least reduce the development of the negative consequences of the burden of care. If support and relief interventions are consistently offered, and if medical help is provided for manifest health problems, using this scale as an instrument for screening for CG burden and estimating the risk of impaired health can have a preventive function and thus possibly even economic advantages for health. An example of a concrete application would be to print the scale in an information brochure along with directions for interpreting the results and concrete recommendations for how to address the problems (see Figs. 2 and 3). For example, such brochures could be distributed to doctors’ practices and counseling centers or made available on the internet.

### Strengths

As the questionnaire was given to all individuals applying for an initial assessment or to upgrade their claims for nursing care benefits by the Bavaria GSHIF, bias due to participant selection was ruled out. The sample could thus be considered representative of informal CGs caring for persons in need of nursing care in Bavaria and covered by statutory insurance. Additional strengths of the sample were its size and the heterogeneity of the CRs (e.g. caregivers of persons with different symptomatologies). The validation was therefore not limited to CGs of persons with dementia as in previously published validation studies on the BSFC [16, 36]. Thus, we were able to show that valid results can also be obtained with the BSFC-s in cases with different causes of care and that it can therefore also be employed in different studies and settings.

The classification system we developed is of special importance. Because it was based on the associations between the percentile ranks of the GBB-24 and the sum score of the BSFC-s, the categories can be considered relevant for the practice of informal nursing care. This is because the norm values used for the GBB-24 were up-to-date and representative of the population. In turn, the uniform interpretation allowed by the classification system permitted a high level of interpretive objectivity. With 10 items, the BSFC-s requires only a very short time to administer. The same applies to the calculation of the results and interpretation.

### Limitations and further research

Because the design of the current study was cross-sectional and not longitudinal, we could not specify the causal directions of the associations. Furthermore, retest reliability could not be determined because there was no second measurement occasion. These weaknesses should be improved in future studies. Whereas persons with different illnesses were included in the study, the age range of the CRs was restricted. Thus, persons with relatives requiring care who were less than 64 years of age were excluded from the study. The use of the scale should be tested in age groups that are not restricted to the care of elderly people. One example might be parents caring for their own children.

## Conclusions

This study has demonstrated that the BSFC-s is a reliable and valid scale for measuring subjective burden in informal CGs. The classification system we developed and the brochures we provide make it easy to use the scale as a screening instrument in various different practice contexts. The risk of negative effects of subjective CG burden can be assessed, and concrete recommendations can be made for how to address it. This can help to prevent the development of additional health impairments in informal CGs and thus possibly also to reduce the costs to health-insurance providers.

## Abbreviations

ADLs: Activities of daily living
BIZA-D: Berlin inventory of caregivers’ burden with dementia patients
BSFC: Burden scale for family caregivers
BSFC-s: Short version of the burden scale for family caregivers
CarerQol: Care-related quality of life instrument
CG: Caregiver
CR: Care recipients
CSI: Caregiver strain index
GBB-24: Giessen subjective complaints list (short form)
GSHIF: Medical Review Board of the German Statutory Health Insurance Funds
IADLs: Instrumental activities of daily living
PHQ-9: Depression module of the patient health questionnaire
PHQ-D: Patient health questionnaire
